# Supplementary material for: Drug Delivery through Epidermal Tissue Cells by Functionalized Biosilica from Diatom Microalgae
Source: Mar Drugs. 2023 Aug 3;21(8):438. doi: 10.3390/md21080438 (PMC10456091; doi:10.3390/md21080438)
Supplement: Supplementary file 1 [file marinedrugs-21-00438-s001.zip › marinedrugs-2524894-supplementary.pdf]

Research Article

Drug delivery through epidermal tissue cells by functionalized biosilica from diatom microalgae

Danilo Vona [1,†], Annarita Flemma [1,†], Francesca Piccapane [2], Pietro Cotugno [1], Stefania Roberta Cicco[3], Vincenza Armenise [1], Cesar Vicente-Garcia [1], Maria Michela Giangregorio [4], Giuseppe Procino [2,*] and Roberta Ragni [1,*]

1.1 *Morphological characterization with optical microscopy*

All functionalized DE samples (DF1, DF5, DF10, DF20 and DF40) were morphologically characterized *via* contrast phase microscopy (transmission modality) in order to highlight possible alteration in the structure of frustules, especially in their meso and nanoporosity. Since the DE utilized in this work was polydisperse, with biosilica shells of different diatom species, observations were done only on valves with a diameter of ~50 μm.

Alkylation seems to not affect the structure of biosilica shells of frustules, as shown in Figure S1. Interestingly, from Figure S1e, the presence of the PDA presence on valves is evident as a general browning coating over the silica lattice of the DE. The coating did not affect the porosity of shells.

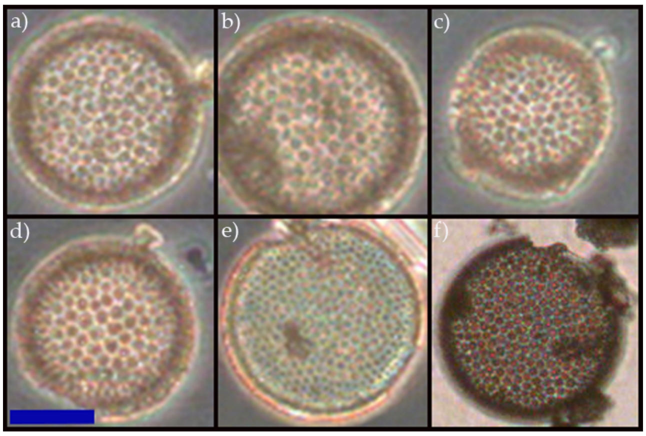

Figure S1. Optical microscopy picture for DF1, DF5, DF10, DF20, DF40 and DF20PDA. Scalebar = 25 μm.

1.2 *Dispersion tests*

Dispersion tests were performed to macroscopically observe and verify the hydrophobic behaviour of functionalized samples. For each sample, 10 mg were dispersed in 5 mL of solvents with different polarities: bi-distilled water, ethanol, methanol, acetone, and hexane. After vigorously shaking, vials were left to settle, and pictures were taken every 10 min for 40 minutes.

Figure S2 shows that DF1, DF5 and DF10 samples have similar behavior, tending to better disperse in polar solvents, meanwhile, in hexane, they quickly sediment in the bottom of vials. Increasing interaction with non-polar solvents is observed starting from DF20: 40 minutes after shaking, DF20 and DF40 are still dispersed in hexane, meanwhile samples in water were partially positioned at the air-water interface or quickly precipitated to the bottom of vials.

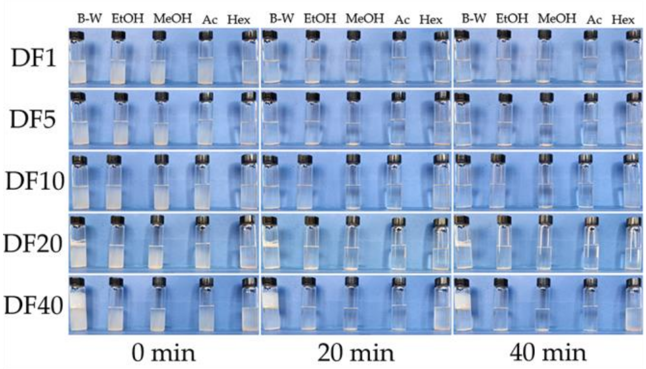

Figure S2. Pictures of dispersion tests for each sample (listed to the left) at 0, 20 and 40 minutes (listed below). Bw = bi-distilled water, Et = ethanol, Me = methanol, Ac = acetone, He = hexane.

1.3 Calibration Curve for Nap determination by fluorescent spectroscopy

In vitro drug delivery was investigated by soaking the drug-loaded diatomite samples (5 mg) in the artificial sweat buffer (10 mL) reported for EN 1811:2011 under continuous stirring. During incubation, 2 mL of buffer was collected every hour, centrifuged, and analysed by an ALT Varian Cary Eclipse fluorimeter, to record the emission peak of Naproxen at 360 nm, as proof of its delivery in solution. Concentrations of released drug amount were evaluated by interpolation with the calibration curve reported in Figure S3, obtained with solutions of Nap dissolved in sweat buffer solution.

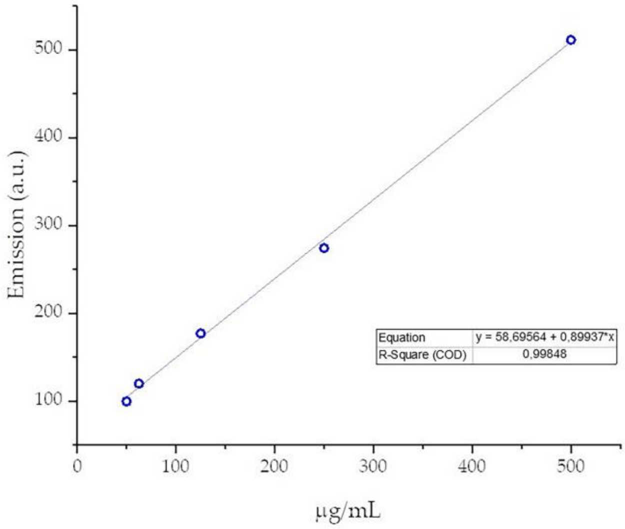

Figure S3. Calibration Curve reporting photoluminescence at 360 nm versus Nap concentration.
